# Simultaneous effective carbon and nitrogen removals and phosphorus recovery in an intermittently aerated membrane bioreactor integrated system

**DOI:** 10.1038/srep16281

**Published:** 2015-11-06

**Authors:** Yun-Kun Wang, Xin-Rong Pan, Yi-Kun Geng, Guo-Ping Sheng

**Affiliations:** 1 CAS Key Laboratory of Urban Pollutant Conversion, Department of Chemistry, University of Science and Technology of China, Hefei, 230026, China; 2 School of Environmental Science and Engineering, Shandong University, Jinan 250100, China

## Abstract

Recovering nutrients, especially phosphate resource, from wastewater have attracted increasing interest recently. Herein, an intermittently aerated membrane bioreactor (MBR) with a mesh filter was developed for simultaneous chemical oxygen demand (COD), total nitrogen (TN) and phosphorous removal, followed by phosphorus recovery from the phosphorus-rich sludge. This integrated system showed enhanced performances in nitrification and denitrification and phosphorous removal without excess sludge discharged. The removal of COD, TN and total phosphorus (TP) in a modified MBR were averaged at 94.4 ± 2.5%, 94.2 ± 5.7% and 53.3 ± 29.7%, respectively. The removed TP was stored in biomass, and 68.7% of the stored phosphorous in the sludge could be recovered as concentrated phosphate solution with a concentration of phosphate above 350 mg/L. The sludge after phosphorus release could be returned back to the MBR for phosphorus uptake, and 83.8% of its capacity could be recovered.

The excess discharge of nitrogen and phosphorus into water bodies results in eutrophication and poor water quality^1^, threatening the health and living environment of human beings^2^. While ironically, resources of these essential nutrients, especially mineable phosphorus, are limited and becoming strategic material for many countries^3^. So, the removal and recovery of these nutrients from wastewater not only have the additional benefits of minimizing eutrophication of water body but also alleviating the potential phosphorus crisis^4^,^5^. For these reasons, phosphate recovery process should be integrated into the existing wastewater treatment plants for nutrient removal^6^,^7^.

Biological approaches have been widely used in wastewater treatment plants for removal of nitrogen and phosphorus^8^,^9^,^10^. By alternating anaerobic-anoxic-aerobic (A^2^O) processes, returning sewage and sludge and discharging the phosphate-rich sludge from the system as excess sludge, simultaneous nitrogen and phosphorus removal can be achieved^11^,^12^. However, such conventional wastewater treatment technologies still present severe technical, economical and sustainability limitations caused by their complex operation and sludge disposal, high energy requirements, poor effluent quality, and no phosphorus recovery process^13^.

In recent years, the MBR process has gained increasing application due to its high treatment efficiency, low sludge production and good effluent quality^14^,^15^,^16^. It has been reported that an aerated MBR can be connected with anaerobic and/or anoxic processes to achieve simultaneous carbon, nitrogen and phosphorus removal during wastewater treatment^17^,^18^,^19^. However, these processes simply connect some individual reactors in sequences, which increase the operation complexity. In recent years, intermittently aerated MBRs have been developed for wastewater treatment and the effect of alternating aeration on/off mode on system performance was studied^20^,^21^,^22^. In our previous study, an intermittently aerated MBR using stainless steel mesh filter was developed to simultaneously remove chemical oxygen demand (COD) and nitrogen under continuous flow mode without any excess sludge discharged^23^. However, the removal of phosphorus was not investigated in that system. To make this technology more practically viable, the configuration and operation of the system need to be further optimized, and the removal and recovery of phosphorus need to be considered. Actually, some strategies for phosphorus recovery from wastewater using modified enhanced biological phosphorus removal^24^,^25^ or biologically induced phosphorus precipitation^26^ could be used for reference in the intermittently aerated MBR.

Herein, an intermittently aerated MBR was further designed to enhance phosphorus recovery from wastewater in this work. The present study investigated the potential of simultaneous removal of COD, TN and TP in a single reactor without excess sludge discharged. Furthermore, the phosphorus recovery was explored. In addition, the mechanism of TN and TP removal in the intermittently aerated MBR were analyzed.

## Results

### Operating performance

**Figure 1:**
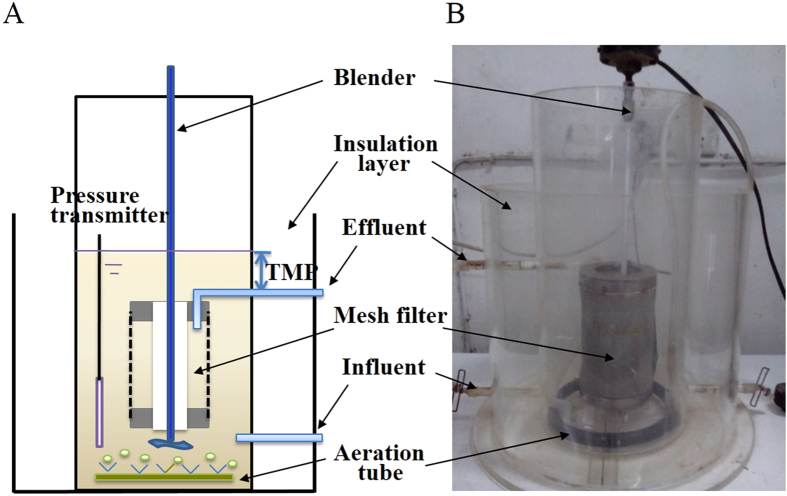
(**A**) Schematic diagram and (**B**) photograph of the MBR system.

In this study, a bench-scale MBR with stainless steel mesh filter was constructed, and the structure is illustrated in Fig. 1. The column-type reactor consists of one aeration tank and one submerged filter module. The tubular hollow mesh filter module was immersed in the reactor to form an internal-loop configuration. A micro-porous aeration tube is equipped at the bottom of the reactor to supply fine air bubbles, while an electric blender is placed under the hollow filter module for fluid mixing during no aeration periods.

**Figure 2:**
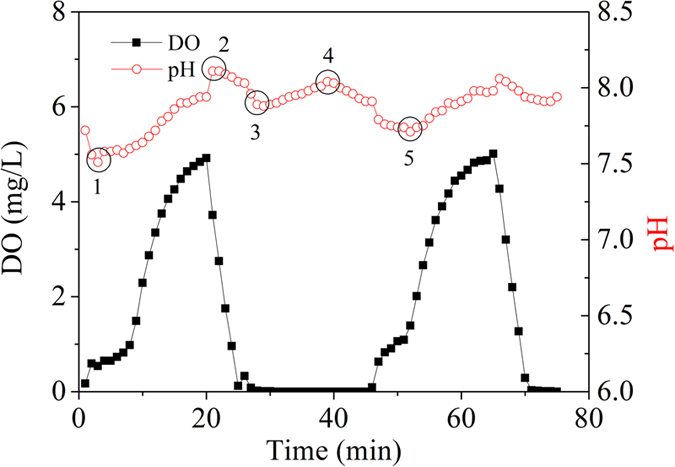
DO and pH changing profiles in a typical aerobic/anoxic cycle.

During the long term operation, the alternating aerobic/anoxic circumstance was provided by periodic switching off aeration. Different biochemical reactions, such as COD heterotrophic biodegradation, nitrification, denitrification, and phosphorus uptake and release by polyphosphate accumulating organisms, occurred in the alternating aerobic/ anoxic cycle, resulting in the changes of DO and solution pH, which are shown in Fig. 2. The DO concentration increased slowly as soon as aeration started, and reached 5 mg/L at the end of aerobic phase, and then dropped rapidly to zero at the initial stage of anoxic phase when the air pump was switched off.

**Figure 3:**
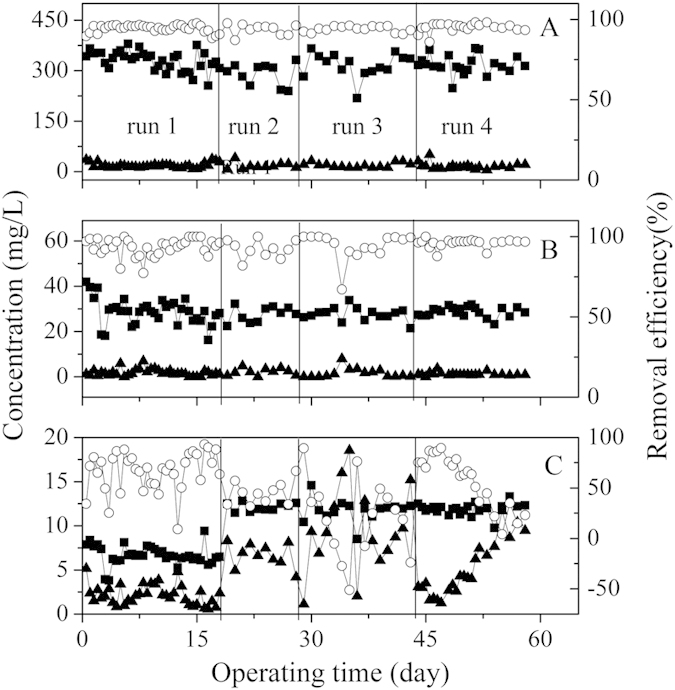
Treatment performance of the reactor with respect to: (**A**) COD; (**B**) TN; (**C**) TP removal. (■) Influent concentration, (▲) effluent concentration and (○) removal efficiency.

The pH changed with the aeration on/off cycles in a similar pattern to the DO changes, but was slightly lagged behind (Fig. 2). The changing of pH resulting from the alternating aerobic/anoxic processes would favor the precipitation of phosphate in sludge^26^,^27^,^28^. Wastewater was continuously fed into the reactor through a peristaltic pump (Lange Co., China) at a constant flow rate (0.5 or 0.67 L/h). The corresponding hydraulic retention time (HRT) was 8 or 6 h, respectively. The system performance in terms of COD, TN and TP at different HRTs and influent concentrations are shown in Fig. 3. And the nutrients removal efficiencies were also summarized in Table 1. The system shows excellent performance in COD and TN removal during the operation. The effluent COD and TN concentrations during the whole experiment period averaged at 17.7 ± 8.3 mg/L and 1.5 ± 1.4 mg/L, respectively. Correspondingly, the COD and TN removal efficiencies averaged at 94.4 ± 2.5% and 94.2 ± 5.7%, respectively.

The TP removal was affected by the influent concentration of phosphorus, HRT as well as the condition of biomass. An average TP removal of 66.4 ± 19.2% was accomplished at a low influent phosphorus of 6.7 ± 1.1 mg/L in run 1. When the influent phosphorus was increased to 12 mg/L in run 2, the TP removal efficiency dropped to 45.1 ± 11.5%. In run 3, the TP removal efficiency further deteriorated and the phosphorus uptake ability of the activated sludge was almost lost, indicating the saturation of the activated sludge for phosphorus uptake. At the end of run 3, the phosphorus-rich sludge was taken out from the reactor for the release of the phosphorus in the sludge. The phosphorus uptake ability of the activated sludge was then partly recovered, and the system performance of COD and TN removal were still high after the sludge was fed back into the MBR (Fig. 3). This result implies that the phosphorus-rich sludge in such an intermittent aerated MBR can be recycled.

### Sludge characteristics in the intermittently aerated MBR

The changes of MLSS and MLVSS in the MBR as well as the TP in biomass over time are shown in Fig. 3. The initial MLSS concentration and the initial MLVSS/MLSS ratio were about 7 g/L and 73%, respectively. During the operation, the MLSS concentration increased slowly while the MLVSS/MLSS ratio decreased slightly, and then maintained at relatively constant levels of 11 g/L and 68%. The MBR had an average COD loading rate of 2.18 kg COD/m^3^/day. A low sludge yield of 0.0275 kg MLSS/kg COD was obtained in this intermittently aerated MBR.

During the experiment, sludge wasting was not conducted due to the low sludge yield, because of a prolonged SRT in the intermittently aerated MBR. At the same time, the excellent retention of sludge by membrane allowed high sludge concentration of sludge, which also contributed to the efficiency nutrients removal and production of high quality effluent water.

**Figure 4:**
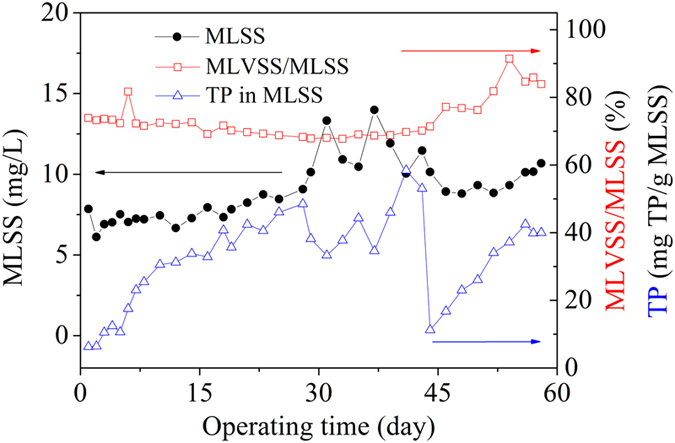
Changes of MLSS, MLVSS and the TP in biomass in the MBR in the experiments.

As shown in Fig. 4, the TP in biomass increased slowly at the initial stage, and then fluctuated around 50 mg TP/g MLSS. When the phosphorus in the sludge was released, the TP in biomass decreased to about 11 mg TP/g MLSS. After the sludge was fed back to the reactor, it increased to about 40 mg TP/ g MLSS during the further operation.

### Phosphorus recovery and sludge recycle

**Figure 5:**
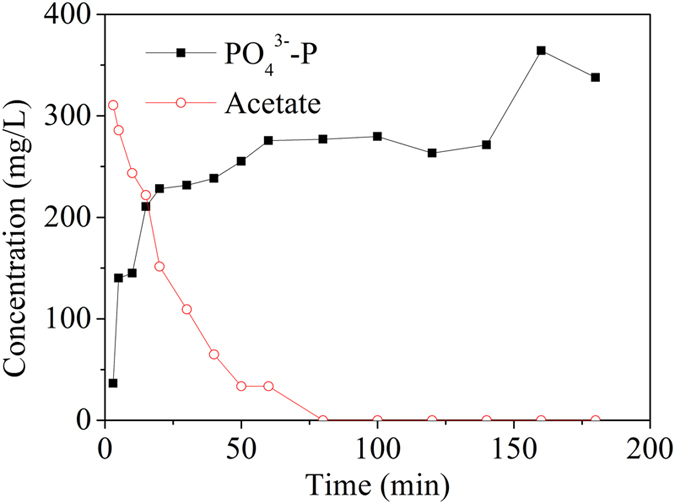
Measurement of phosphate and acetate during anaerobic batch tests with acetate addition performed on day 43.

The activated sludge in the MBR could uptake phosphorus from supernatant, and stored it in biomass. In order to determine the phosphorus release capacity of polyphosphate accumulating organisms (PAOs) and phosphorus uptake performance of the activated sludge after phosphorus releasing, the sludge in the MBR was taken out from the reactor on day 43, then mixed with concentrated extra carbon source, sodium acetate. As shown in Fig. 5, phosphorus was quickly released as soon as the acetate added into the sludge, and the concentration of orthoP in the supernatant increased to about 364 mg/L. It implies that about 70% of phosphorous in the activated sludge was recovered by comparing the total phosphorus in biomass before releasing.

The feasibility of phosphorus enrichment and sludge recycle has been demonstrated in this study. About 68.7% phosphorus could be released from the sludge. After phosphorus release, the biomass was retuned back to the MBR, and its phosphorus uptake performance was evaluated (run 4). As shown in Fig. 3 and Table 1, the recycled sludge still exhibited excellent COD and TN removal performance. At the same time, about 58.3 ± 25.6% of phosphorus could be removed. After 15-days operation in run 4, the total amount of phosphorus in biomass increased to 1.16 g, accounting for 69.5% of the amount of phosphorus release. This result suggests that most of phosphorus uptake capacity of the recycled sludge could be recovered.

### Bioactivities of biomass for simultaneous nitrogen and phosphorus removal

**Figure 6:**
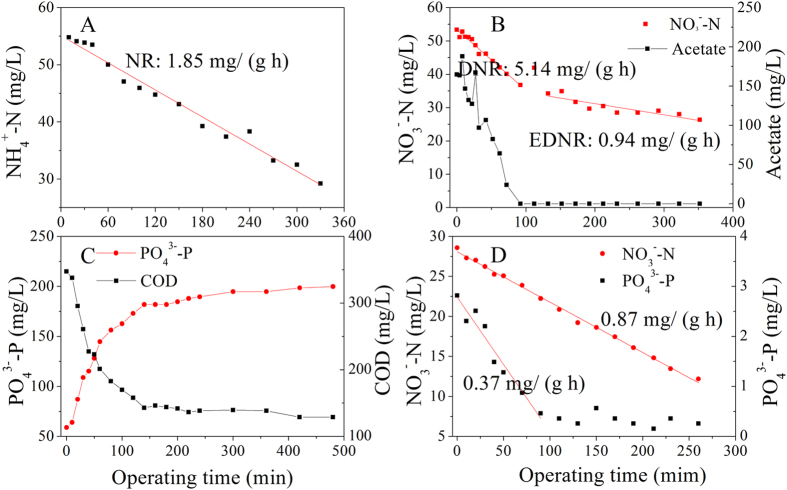
The bioactivities of sludge at Day 43 in terms of: (**A**) nitrification; (**B**) denitrification; (**C**) anaerobic phosphorus release and (**D**) anoxic denitrification phosphorus uptake rate.

In order to analyze the mechanism of nitrogen and phosphorus removal in the intermittently aerated MBR, the bioactivities of sludge for COD, TN and TP removal were measured at the end of run 3. As shown in Fig. 6A,B, the nitrification rate (NR) and denitrification rate (DNR) of the sludge samples were 1.85 mg N/g MLVSS/h and 5.14 mg N/g MLVSS/h respectively, implying high bioactivities of the sludge for nitrogen removal. When acetate was consumed, the DNR decreased to 0.94 mg N/g MLVSS/h, which was termed as endogenous denitrification rate (EDNR). The high bioactivities of the sludge for nitrogen removal and high sludge concentration as well as plenty of nitrifier and denitrifier (Figure S1) in the intermittently MBR also contributed to the high nitrogen removal.

In this intermittently aerated MBR, PAOs including denitrifying PAOs played an important role in phosphorus removal. Figure 6C showed the phosphorus release curve under anaerobic condition. The COD was taken fast with the release of the PO_4_^3-^-P. The sludge sample after phosphate release was then used to determine the anoxic denitrification phosphorus uptake rate of PAOs. Figure 6D showed the variation of the PO_4_^3-^-P and NO_3_^-^-N concentrations under anoxic conditions. Both the PO_4_^3-^-P and NO_3_^-^-N concentrations decreased immediately as soon as nitrate was added, implying that the denitrifying phosphorus uptake occurred under anoxic conditions using nitrate as the electron acceptor instead of oxygen to oxidize poly- β-hydroxybutyrate. So, there were two pathways for phosphorus removal in the intermittently aerated MBR. In aerobic phase, external phosphorus could be assimilated by PAOs for polyphosphate recovery. In anoxic phase, DPAOs with an enhanced phosphorus uptake with significance COD saving, would contribute for both nitrogen and phosphorus removal. Previous studies have reported that the use of nitrate instead of oxygen as electron acceptor to simultaneously remove phosphorous and nitrogen from wastewater leads to a lower sludge yield as well as the efficient removal of COD^29^,^30^.

### Biofilm formation during the long-term operation

**Figure 7:**
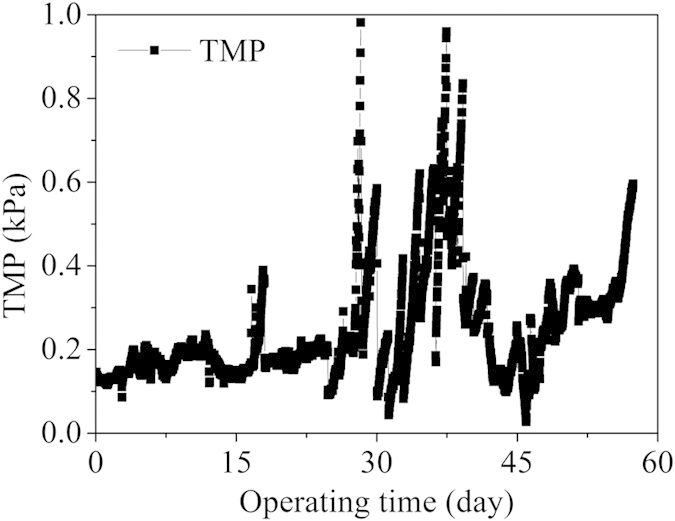
Variation of water head drop during the long-term operation.

The effluent turbidity was monitored during most of the operating period. The low effluent turbidity indicated the good retention of the biomass by the mesh filter in the reactor (data not shown). In this mesh filter MBR, the filtration process was driven by gravity. During operation period, the permeation fluxes of the MBR were kept around 20–27 L/m^2^/h. As shown in Fig. 7, the TMP increased gradually with the slowly development of biofilm on mesh in the initial period. However, after a long-term operation, the biofilm attached on steel mesh become thick, and the TMP increase sharply. When it reached about 1 kPa, off-line backwashing was periodically carried out to remove the overgrown biofilm, and the TMP dropped immediately after each backwashing. After the initial cleaning, the TMP decreased mostly to its initial level, however, more frequently mesh cleaning was needed at the later stage of the operation, implying that irreversible fouling would occur after long-term operation.

## Discussion

Simultaneous removal of COD, TN and TP in a single reactor is favorable for the wastewater treatment due to its economic benefit and environmentally friendliness^31^,^32^. Herein, an intermittently aerated membrane bioreactor with a stainless steel mesh filter was developed for simultaneous pollutants removal and nutrient recovery, with special emphasis on phosphorus recovery from wastewater. Concentrated phosphate stream was released from the phosphorus-rich sludge by adding a few extra carbon source. Most importantly, the sludge was recycled and there was no excess sludge discharged during the experiments, which means the cost of sludge disposal could be saved. Furthermore, the intermittent aeration operation is promising to reduce energy consumption in MBRs. In this study, with aeration on/off time ratio of 25 min/20 min, about 44% aeration energy could be reduced compared with the continuously aerated systems.

In this study, the intermittently aerated MBR showed higher COD, TN and TP removal efficiencies compared to other biological nutrients removal systems. For nitrogen removal process, complete nitrification was achieved during the most of the experimental periods, while denitrification was enhanced by inserting an anoxic phase in the system. For phosphorus removal process, external phosphorus could be assimilated by PAOs in aerobic phase, while denitrifying PAOs would contribute for both nitrogen and phosphorus removal in anoxic phase. The denitrifying PAOs process is capable of performing simultaneous denitrification and anoxic phosphorus uptake and advantageous in saving aeration, reducing the demand for external carbon sources and minimizing sludge yield. Our experiment results showed that the denitrifying phosphorus uptake might occur using nitrate as the electron acceptor instead of oxygen in the intermittently MBR (Fig. 6D). The presence of PAOs dominated the intermittently aerated MBR biomass was confirmed by the FISH images (Figure S1), which might perform the denitrification activities^33^. In our future research, it is necessary to investigate the contribution of denitrifying PAOs on phosphate removal in the intermittently MBR.

The alternating anoxic/aerobic processes in the intermittently aerated MBR resulted in high microbial activities of nitrifying bacteria, denitrifying bacteria and PAOs in the MBR. The results suggest that metabolic selection via alternating anoxic/aerobic processes has a potential to enhance bioactivities and thus improve nitrogen and phosphorus removal in the intermittently aerated MBR. The excellent retention of sludge by membrane allowed high sludge concentrations, which also contributed to improve the slow-growing microorganisms and thus improve the nitrifying and denitrifying activities in the MBR.

Just as our previous reports^34^,^35^,^36^, utilization of cheap stainless steel mesh in this study, as an alternative to conventional microfiltration/ultrafiltration membrane can lower the MBR construction costs, increase the economical viability and promote the application of such processes^37^,^38^. In this mesh filter MBR, the filtration process was driven by gravity. The energy demand for draining water as well as membrane fouling in the modified MBR be reduced compared with conventional MBR^39^.

The modified MBR system integrates both nutrient removal and phosphorus recovery, which presents a promising system for wastewater treatment and resource recovery. In this study, the removal of total phosphorus in a modified MBR was averaged at 53.3 ± 29.7%. The removed TP from wastewater was stored in biomass, and 68.7% of the stored phosphorous in the sludge could be recovered as highly concentrated phosphate solution, whose concentration was about 30 times more than that in the wastewater, and was sufficient for chemical precipitation^40^. Higher phosphate concentrations would lead to more effective phosphorus recovery, i.e., directly using as fertilizer, or precipitating as magnesium ammonium phosphate (struvite)^41^. If such a strategy is adopted for a wastewater treatment plant with a capacity of 50,000 m^3^/day and an influent phosphorus concentration of 10 mg/L, amount of about 267 kg/day phosphorus could be removed from the influent wastewater, and almost 200 kg/day phosphorus could be enriched as concentrated phosphate solution, accompanied with production of high quality reclaimed water. In this study, the system performances were evaluated and the mechanism of simultaneous nitrogen and phosphorus removal was investigated. Nevertheless, to promote the practical application of this technology, the phosphorus removal efficiency as well as phosphorus recovery capacity of the MBR system should be further improved.

## Materials and Methods

### Reactor setup

In this study, a bench-scale MBR with stainless steel mesh filter (Huayang Ironware Co., China) was constructed, and the structure is illustrated in Fig. 1. The column-type reactor has a working volume of 4 L and consists of one aeration tank and one submerged filter module. The tubular hollow mesh filter module, with an average pore size of 53 μm and an effective filtration area of 0.025 m^2^, was immersed in the reactor to form an internal-loop configuration. A micro-porous aeration tube is equipped at the bottom of the reactor to supply fine air bubbles, while an electric blender is placed under the hollow filter module for fluid mixing during no aeration periods.

### Inoculation and operation conditions

The aeration tank was seeded with activated sludge from an enhanced biological phosphorus removal reactor. Initial mixed liquor suspended solids (MLSS) concentration was 7.85 g/L. No excess sludge was discharged during the entire experimental period except for sample analysis.

Synthetic wastewater was used, which contained CH_3_COONa·3H_2_O, NH_4_Cl, and K_2_HPO_4_·3H_2_O as the sources of carbon, nitrogen, and phosphorus, respectively. The COD, NH_4_^+^-N and PO_4_^3-^-P concentrations in the influent during the experiments are listed in Table 1. Other trace elements were added into the synthetic wastewater. The composition of the trace element solution (in μg/L) was: EDTA, 50, ZnSO_4_·7H_2_O, 22, CaCl_2_·2H_2_O, 8.2, MnCl_2_·4H_2_O, 5.1, FeSO_4_·7H_2_O, 5.0, (NH_4_)_6_Mo_7_O_24_·4H_2_O, 1.1, CuSO_4_·5H_2_O, 1.8, CoCl_2_·6H_2_O, 1.6. Wastewater was continuously fed into the reactor through a peristaltic pump (Lange Co., China) at a constant flow rate (0.5 or 0.67 L/h). The corresponding hydraulic retention time (HRT) was 8 or 6 h, respectively. The temperature was controlled at 25 ± 1 ^o^C during the experiment. The trans-membrane pressure (TMP) across the mesh, reflected by the water head drop, was online monitored using a pressure transmitter (LD187, Leide Electronic Ltd., China). The TMP would increase when the biofilm attached on mesh became thick. Once the TMP reached 1.0 kPa, off-line backwashing was carried out to remove the overgrown biofilm. Intermittent aeration was conducted, with the aerobic/anoxic period ratio of 20 min /25 min, which was automatically controlled by a time relay (Xinling Electrical Co., Ltd. China). During the anoxic, the reactor was continuously stirred using an electric blender.

The recovery of phosphorus from the sludge and phosphorus uptake capacity of the recycled sludge over time was evaluated. On day 43, the sludge in the MBR was all taken out, washed five times with tap water to remove the residual COD, NH_4_^+^-N, NO_3_^-^-N, and then mixed with concentrated sodium acetate and diluted to a volume of 4.6 L to release phosphorus. After 3 hours mixing, the sludge was centrifuged at 7100 × g for 3 min to remove supernatant. The collected sludge after phosphorus release was fed back to the MBR for phosphorus removal.

### Analysis and calculations

MLSS, mixed liquor volatile suspended solids (MLVSS), COD, TN, TP concentrations and turbidity were measured following the Standard Methods^42^. The measurements of NH_4_^+^-N, NO_3_^-^-N, PO_4_^3-^-P were conducted using a water quality autoanalyzer (Aquakem 200, ThermoFisher, Finland) according to the standard methods. The DO concentration and pH were monitored with DO meter (HQ 30d, Hach Co., USA) and pH meter (PHS-3C, INESA Scientific Instrument Co., Led, China).

The bioactivities of sludge in terms of specific nitrification rate (SNR), specific denitrification rate (SDNR), anaerobic phosphorus release rate and phosphorus uptake rate under anoxic conditions were investigated. The activated sludge samples were taken from the intermittently aerated MBR at Day 43 for determining nitrification, denitrification and anaerobic phosphorus release bioactivities of sludge. Nitrogen gas was purged through each reactor to maintain anaerobic or anoxic conditions, when necessary.

To determine SNR, the sludge sample was spiked with ammonium chloride to a concentration of approximately 55 mg/L NH_4_^+^-N. During this batch experiment, the DO level was controlled at about 5 mg/L by switching on/off of the aeration pump. To measure the denitrification activity of the activated sludge in the system, SDNR was determined following the method described in a previous study^43^. To measure the anaerobic phosphorus release rate, acetate was dosed into the reactor to a final equivalent COD concentration of 350 mg/L at initial. After complete releasing of phosphate, the sludge was centrifuged and washed to remove the residual COD and phosphate. Then, NaNO_3_ and K_2_HPO_4_·3H_2_O were added into the sludge sample under anoxic conditions to determine the phosphorus uptake rate of denitrifying polyphosphate accumulating organisms.

## Additional Information

**How to cite this article**: Wang, Y.-K. *et al.* Simultaneous effective carbon and nitrogen removals and phosphorus recovery in an intermittently aerated membrane bioreactor integrated system. *Sci. Rep.* **5**, 16281; doi: 10.1038/srep16281 (2015).

## Supplementary Material

Supporting Information

## Figures and Tables

**Table 1:** System performance during different experimental period.

Run	Operation time (day)	HRT (h)	Inflow (mg/L)			Effluent (mg/L)			Removal efficiency (%)		
			COD	TN	TP	CO	TN	TP	COD	TN	TP
1	1–18	8	330.2 ± 29.2	28.8 ± 5.8	6.7 ± 1.1	18.3 ± 7.6	1.8 ± 1.5	2.2 ± 1.1	94.4 ± 2.3	93.6 ± 5.5	66.4 ± 19.2
2	19–28	8	290.0 ± 33.1	27.6 ± 3.4	12.1 ± 0.5	17.6 ± 9.7	2.2 ± 1.5	6.7 ± 1.4	93.8 ± 3.4	92.2 ± 5.6	45.1 ± 11.5
3	29–43	6	316.7 ± 36.7	27.6 ± 2.9	11.7 ± 1.3	19.2 ± 7.2	1.2 ± 1.2	9.6 ± 4.8	94.0 ± 1.8	94.2 ± 8.6	20.4 ± 38.5
4	44–58	6	318.0 ± 27.6	28.7 ± 2.2	11.9 ± 0.7	15.8 ± 9.6	1.1 ± 0.7	5.0 ± 3.1	95.0 ± 1.7	96.0 ± 2.7	58.3 ± 25.6

Note: the nutrient concentrations in inflow and effluent as well as the removal efficiencies were calculated as the average values during the whole periodic experiments.
