# Supplementary material for: Simultaneous effective carbon and nitrogen removals and phosphorus recovery in an intermittently aerated membrane bioreactor integrated system
Source: Sci Rep. 2015 Nov 6;5:16281. doi: 10.1038/srep16281 (PMC4635345; doi:10.1038/srep16281)
Supplement: Supporting Information [file srep16281-s1.doc]

**Supporting information:**

**Simultaneous effective carbon and nitrogen removals and phosphorus recovery in an intermittently aerated membrane bioreactor integrated system**

Yun-Kun Wang,^1,2^ Xin-Rong Pan,^1^ Yi-Kun Geng,^1^ Guo-Ping Sheng^1^*

^1^CAS Key Laboratory of Urban Pollutant Conversion, Department of Chemistry, University of Science and Technology of China, Hefei, 230026, China

^2^School of Environmental Science and Engineering, Shandong University, Jinan 250100, China

* **Corresponding author**:

Dr. Guo-Ping Sheng, Fax: +86-551-63601592, E-mail: gpsheng@ustc.edu.cn

**Fluorescence in situ hybridization (FISH).** Biomass (3 mL culture liquid) was harvested at the end of run 3 from the intermittently aerated membrane bioreactor (MBR), centrifuged, washed with phosphate-buffered saline (PBS; 10 mM Na_2_HPO_4_/NaH_2_PO_4_ pH 7.2 and 130 mM NaCl) and fixed for 1-3 h with 4% (w/v) paraformaldehyde in PBS on ice. After washing with PBS, samples were stored in PBS/ethanol (1:1) at -20°C.

For microscopy, fixed biomass was spotted in 10 μL volumes onto the wells of microscope slides. After sequential dehydration in 50%, 80% and 95% ethanol for 3 min, each probe was hybridized for 1.5 h at 46°C in hybridization buffer containing 900 mM NaCl, 20 mM Tris/HCl pH 8.0, 0.1‰ sodium dodecyl sulfate, 50% formamide and a combination of the following oligonucleotide probes: NSO190 and NSO1225 (AOB) [^1^](#_ENREF_1), (NIT3 & NSR1156) (NOB) [^1^](#_ENREF_1), EUB 338 (most Bacteria) [^2^](#_ENREF_2), and CY3-PAO651 (PAO) [^3^](#_ENREF_3).

For image acquisition, an OLYMPUS EX51 epifluorescence microscope equipped with a CCD camera was used together with the DP2-BSW software package (OLYMPUS, Japan).


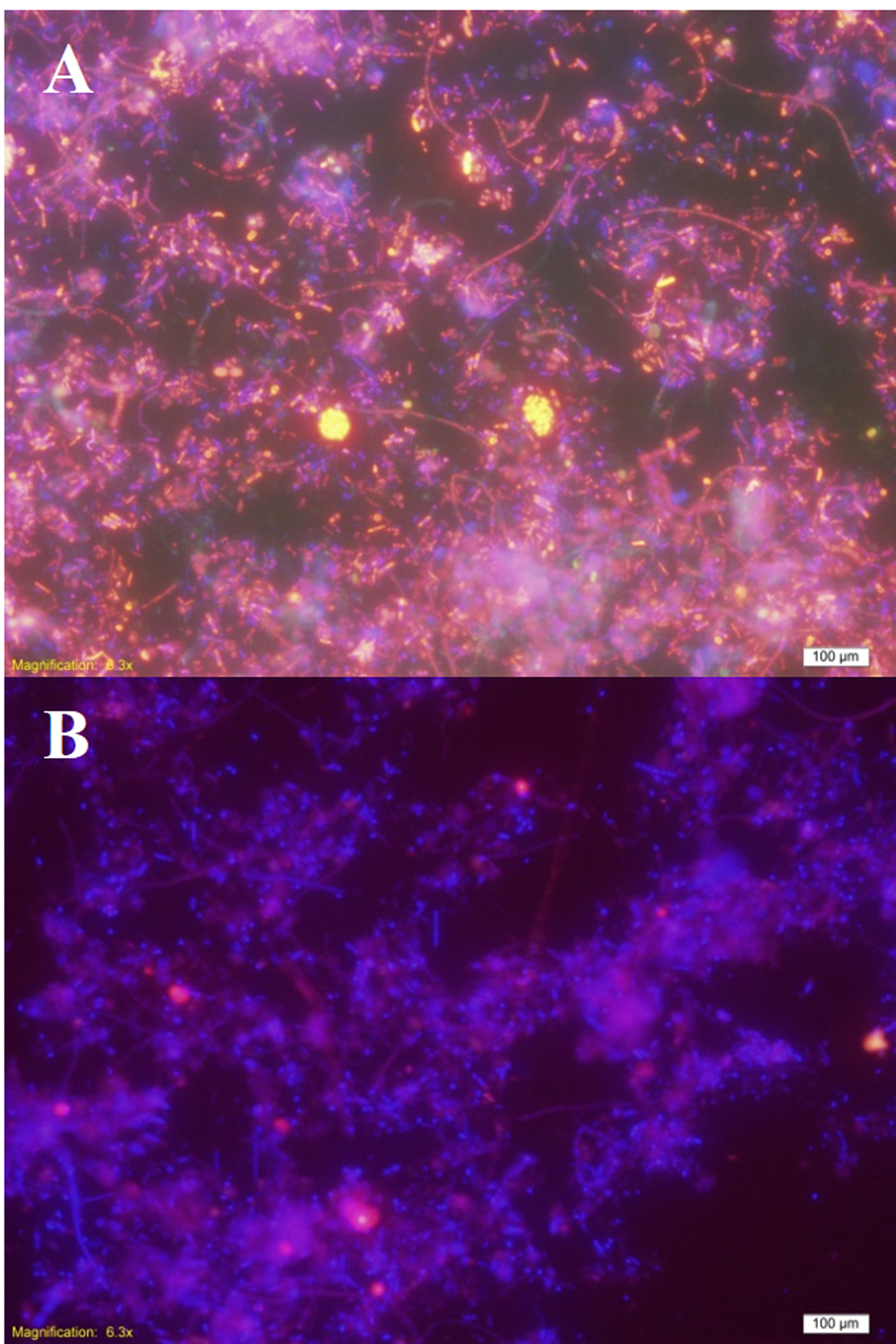


**Figure S1.** FISH images of the biomass: (A) red-all bacteria; blue-AOB; green-NOB (B) blue-all bacteria; red-PAO.

**References**

1. Gieseke, A., Purkhold, U., Wagner, M., Amann, R. & Schramm, A. Community structure and activity dynamics of nitrifying bacteria in a phosphate-removing biofilm. *Appl. Environ. Microbiol.* **67**, 1351-1362 (2001).

2. Daims, H., Brühl, A., Amann, R., Schleifer, K. H. & Wagner, M. The domain-specific probe EUB338 is insufficient for the detection of all bacteria: Development and evaluation of a more comprehensive probe set. *Syst. Appl. Microbiol.* **22**, 434-444 (1999).

3. Wang, J. F., Zhao, Q. L., Jin, W. B., You, S. J. & Zhang, J. N. Performance of biological phosphorus removal and characteristics of microbial community in the oxic-settling-anaerobic process by FISH analysis. *J. Zhejiang Univ. Sci. A* **9**, 1004-1010 (2008).
